# Supplementary material for: Stochastic fluctuations in apoptotic threshold of tumour cells can enhance apoptosis and combat fractional killing
Source: R Soc Open Sci. 2020 Feb 19;7(2):190462. doi: 10.1098/rsos.190462 (PMC7062090; doi:10.1098/rsos.190462)
Supplement: Mathematical derivations and supplementary information. [file rsos190462supp1.docx]

**S1 Supporting Information**

**Stochastic fluctuations in apoptotic threshold of tumor cells can enhance apoptosis and combat fractional killing**

Baohua Qiu^1,2^, Jiajun Zhang^1,2*^, Tianshou Zhou^1,2*^

1. Guangdong Province Key Laboratory of Computational Science, Sun Yat-Sen University, Guangzhou, People's Republic of China.
2. School of Mathematics, Sun Yat-Sen University, Guangzhou, People's Republic of China

In this supplement, we provide details for mathematical derivations in the main text and present figures that illustrates some of the results in greater depth.

**Contents**

[1 Mathematical modeling for FPT problem 2](#_Toc22820090)

[1.1 A FPT problem with fluctuating threshold 2](#_Toc22820091)

[1.2 Probability density function of FPT 3](#_Toc22820092)

[1.3 Statistical quantities of FPT 5](#_Toc22820093)

[2 Gene expression model of FPT with fluctuating threshold 6](#_Toc22820094)

[3 FPT distribution and its statistics in four different fluctuating thresholds 8](#_Toc22820095)

[3.1 The absorbing domain is 9](#_Toc22820096)

[3.2 The absorbing domain is 14](#_Toc22820097)

[3.3 The absorbing domain is 17](#_Toc22820098)

[3.4 The absorbing domain is 21](#_Toc22820099)

[4 Effect of timescales on the timing event 25](#_Toc22820100)

[5 FPT statistics in the case that burst size follows Poisson distribution 26](#_Toc22820101)

[6 FPT problem involved cell division 30](#_Toc22820102)

[References 30](#_Toc22820103)

# 1 Mathematical modeling for FPT problem

## 1.1 A FPT problem with fluctuating threshold

Let $\left\{p53:p53=p53\left(t\right),t\geq0\left|p53\left(0\right)=p53_{0}\right.\right\}$ be a temporally homogeneous diffusion process, and $\left\{A:A=A\left(t\right),t\geq0\left|A\left(0\right)=A_{0}\right.\right\}$ be a fluctuating barrier (called the barrier system). Without loss of generality, we set or . This setting is natural since represents a boundary or threshold that will cross. Note that the union of and ,$\left\{\left.\left(p53,A\right):p53=p53\left(t\right),A=A\left(t\right),t\geq0\right|p53\left(0\right)=p53_{0},A\left(0\right)=A_{0}\right\}$ constitutes a new system or a new process. Define as the time thathits the fluctuating barrierfor the first time, that is ([1](#refs1), [2](#refs2), [3](#refs3)),

$T=\min\left\{t:\left.p53\left(t\right)\geq A\left(t\right)\right|p53\left(0\right)=p53_{0}<A\left(0\right)=A_{0}\right\}$ (S1)

which is called the first passage time (FPT). Apparently, is a random variable since both and are random, referring to [Fig. S1*A*](#figs1), and depends on and .

**Fig. S1** **Event timing is modeled as a problem of first passage time (FPT)**. (A) shown is an example for FPT, where represents the boundary and the solid circle represents the point that stochastic trajectory hits stochastic trajectory for the first time; (B) an absorbing domain of PFT, defined by . Note that the timing event is triggered once reaches .

To describe the theoretical frame of FPT problem , we here define an absorbing domain , which consists of those points $p53,A()$ that satisfies in the $p53,A()$ plane, that is, (referring to Fig. S1(*B*)), and define a survival probability, , that the trajectory starting from at time has not yet been absorbed into domain at time $t$. Assume that $P_{X',Y'}\left(t\right)$ represents the probability that the system $\left\{\left(p53,A\right)\text{：}p53=p53\left(t\right),A=A\left(t\right),t\geq0\right\}p53\left(0\right)=p53_{0}<A\left(0\right)=A_{0}\{\}$ is at state $X',Y'()$ at time , that is,

(S2)

For convenience, $P_{X',Y'}\left(t\right)$ is sometimes denoted by $P_{S'}\left(t\right)$, i.e., $P_{S'}\left(t\right)=P_{X',Y'}\left(t\right)$, where $S'=\left(X',Y'\right)$ represents state. Note that the survival probability can be expressed as

. (S3)

Denote by $f_{T}(t)$ the probability density function of the FPT, that is,

$f_{T}(t)=\text{Prob}\left\{T\leq t\right\}$. (S4)

In the following, we are mainly interested in statistical properties of random variable $T$. For this, we first establish the relation between $f_{T}(t)$ and , and then give the expression of $f_{T}(t)$.

## 1.2 Probability density function of FPT

Assume that all states with constitute a Markov process. Then, we have a forward master equation (FME) of the form

(S5)

where is a column vector consisting of all $P_{S}\left(t\right)$, and is a certain linear operator, depending a process of interest. Note that every component of is the probability that the system arrives at the absorbing domain at time , and that is actually a state transition matrix, whose components can be expressed as

(S6)

where $\alpha\left(S,S'\right)$ denotes the transition rate from state $S'$ to state $S$, and $\delta_{S,S'}$ is the Kronecker delta function.

On the other hand, there is an absorbing state $S_{f}$ with $S_{f}\in\left\{\left(p53_{f},A_{f}\right)\right\}$, such that the transition rate from absorbing state $S_{f}$ to state $S$ is always zero, that is, $\alpha\left(S,S_{f}\right)=0$ for all states $S$. According to the definition of , we can know the conservative condition: . Let $P\left(\left.S_{f},t\right|S,t_{0}\right)$ be the probability that the state reaches the absorbing state $S_{f}$ at time $t$, given the initial state at time with (usually, is set). Then, we can determine the probability density function, $f\left(t;\left.S_{f}\right|S\right)$, of the FPT for state $S$ to reach absorbing state $S_{f}$.

Denote by the survival probability that the trajectory starting from at time has not yet been absorbed to state $S_{f}$ at time $t$, that is,

(S7)

By definition, we can have

. (S8)

In fact, the probability that state $S$ reaches state $S_{f}$ in an infinitesimal time interval $\left(t,t+dt\right)$ is $f\left(t;\left.S_{f}\right|S\right)dt$, so the probability that state $S$ reaches state $S_{f}$ at time $t$ is given by $\int_{0}^{t} f\left(t';\left.S_{f}\right|S\right)dt'$. Thus, the survival probability is related to the FPT distribution through the following manner

(S9)

In turn, we can determine the probability density function of the FPT. In fact, it follows directly from [Eq. S9](#eqs9) that

(S10)

Using [Eq. S5](#eqs5) and [Eq. S7](#eqs7), we further have ([1](#refs1), [2](#refs2))

(S11)

where we have used the fact of due to the probability conservation. Thus, the probability density function of the FPT is given by

(S12)

which can simply be expressed as the following vector form

$f_{T}\left(t\right)=W^{\text{T}}\mathbf{P}\left(t\right)$ (S13)

Note that [Eq. S13](#eqs13) has the solution of the form

$f_{T}\left(t\right)=W^{\text{T}}\exp\left(\mathbf{M}t\right)\mathbf{P}\left(0\right)$ (S14)

where $W$ is the column vector of the transition rates from all accessible states $S''$ to state $S_{f}$.

## 1.3 Statistical quantities of FPT

Once the probability density function of the FPT $f_{T}\left(t\right)$ is given, we can calculate raw moments of random variable $T$, according to

, (S15)

Substituting the expression of $f_{T}\left(t\right)$ into the above [Eq. S15](#eqs15), and using the integration by parts for many times, can be transformed the following form

$\begin{matrix} \left\langle T^{k}\right\rangle=W^{\text{T}}\left[\int_{0}^{\infty} t^{k}\exp\left(\mathbf{M}t\right)dt\right]\mathbf{P}\left(0\right) \\ =W^{\text{T}}\left[\left.t^{k}\mathbf{M}^{-1}\exp\left(\mathbf{M}t\right)\right|_{0}^{\infty}-k\mathbf{M}^{-1}\int_{0}^{\infty} t^{k-1}\exp\left(\mathbf{M}t\right)dt\right]\mathbf{P}\left(0\right) \\ =\left(-1\right)^{k+1}k!W^{\text{T}}\left(\mathbf{M}^{-1}\right)^{k+1}\mathbf{P}\left(0\right) \end{matrix}$ (S16)

where the characteristic values of matrix $\mathbf{M}$ are assumed to have negative real parts, and $t^{k}\mathbf{M}^{-1}\exp\left(\mathbf{M}t\right)\text{ }\left(k=0,1,2,\ldots\right)$ in the above expression goes to zero. Moreover, $W^{\text{T}}\mathbf{M}^{-1}=-\mathbf{e}^{\text{T}}$ can be obtained due to $\left\langle T\right\rangle\equiv1$, where $e^{\text{T}}=\left[1,1,\cdots,1\right]$ is a constant vector. Thus, we easily show

(S17)

Furthermore, it follows from [Eq. S17](#eqs17) that the mean FPT, i.e., timing mean (MFPT), of the probability density function $f_{T}(t)$ and the intensity of the noise with $T$ of FPT, (defined as the ratio of variance over the square of mean), denoted by timing variability ($CV_{T}$), are given by

(S18)

(S19)

In addition, we can calculate the high order features, such as skewness and kurtosis, according to calculation formula,

(S20)

(S21)

We can give the explicit expressions of skewness and kurtosis, based on [Eq. S17](#eqs17).

# 2 Gene expression model of FPT with fluctuating threshold

From now on, we consider a common model of stochastic gene expression with a stochastically fluctuating threshold, referring to [Fig. S2](#figs2) and S3*A*. A threshold event is triggered once the expression level of a gene, denoted by $p53$, crosses the expression level of another gene, denoted by $A$ (as a critical threshold) for the first time. We are interested in the stochastic but not deterministic threshold crossing.

**Fig. S2** **Schematic for showing two gene expressions mode**, where the gene p53 is expressed in a burst manner whereas the gene A is expressed in a constitutive manner.

Assume that the $p53$ molecules are produced in a burst manner whereas the $A$ molecules are generated in a constitutive manner. We use the produced counts of protein $A$ to construct a stochastically fluctuating threshold that the molecule number of protein $p53$ reaches. Let $A\left(t\right)\in\left\{0,1,2,\cdots\right\}$ denote the level of protein $A$ at time $t$, and assume that $A\left(t\right)$ follows a Poisson distribution with two characteristic parameters $g_{\text{A}}^{(n)},$ (where superscript $(n)$ means that feedback regulation is considered. This superscript may be omitted in the absence of feedback regulation) and $d_{\text{A}}$ representing respectively the transcription and degradation rates of protein $A$ when $A\left(t\right)=n$. Let $p53\left(t\right)\in\left\{0,1,2,\cdots\right\}$ denote the level of protein $p53$ at time $t$, and assume that protein $p53$ is generated with a Poisson rate $g_{\text{p53}}^{\left(m\right)}$ (where superscript $(m)$ means that feedback regulation is considered, and it may be neglected in the absence of feedback regulation), and degrades at a constant degradation rate $d_{\text{p53}}$. The translation burst approximation is based on the assumption of short-lived mRNAs, that is, each mRNA degrades instantaneously after producing a burst of $B$protein molecules, where $B$ is assumed as usual that follows geometric distribution

$P_{B}\left(B=k\right)=\frac{b^{k}}{\left(1+b\right)^{k+1}},k=0,1,2,\cdots,b>0$ (S22)

Then, we can show

$P_{B}\left(B\geq k\right)=\left(\frac{b}{1+b}\right)^{k},k=0,1,2,\cdots$ (S23)

where $b$ represents the mean protein burst size. In what follows, we denote $P_{B=k}\equiv P_{B}\left(B=k\right)$ and $P_{B\geq k}\equiv P_{B}\left(B\geq k\right)$ for convenience.

The time evolution of $p53\left(t\right),A\left(t\right)()$ starting from $p53\left(0\right)=m,A\left(0\right)=n$ with $m<n$ at time $t=0$ can be descried through the following probabilities of timing events in the next infinitesimal time $\left(t,t+dt\right)$

$\begin{matrix} P\left(\left.p53\left(t+dt\right)=m+B,A\left(t+dt\right)=n\right|p53\left(t\right)=m,A\left(t\right)=n\right)=g_{\text{p53}}^{\left(m\right)}dt; \\ P\left(\left.p53\left(t+dt\right)=m-1,A\left(t+dt\right)=n\right|p53\left(t\right)=m,A\left(t\right)=n\right)=md_{\text{p53}}dt; \\ P\left(\left.p53\left(t+dt\right)=m,A\left(t+dt\right)=n+1\right|p53\left(t\right)=m,A\left(t\right)=n\right)=g_{\text{A}}^{\left(n\right)}dt; \\ P\left(\left.p53\left(t+dt\right)=m,A\left(t+dt\right)=n-1\right|p53\left(t\right)=m,A\left(t\right)=n\right)=nd_{\text{A}}dt; \end{matrix}$ (S24)

An event occurs if the cumulative number of proteins reaches the number of protein molecules. The relation between the proteins and can be considered as a trajectory in the domain . The corresponding forward master equation (FME) describing the time evolution of protein pair and can be described as [[4](#refs4)]

$\begin{matrix} \frac{dP_{m,n}\left(t\right)}{dt}=\sum_{i=0}^{m-1} g_{\text{p53}}^{(i)}P_{B}\left(B=m-i\right)P_{i,n}\left(t\right)+g_{\text{A}}P_{m,n-1}\left(t\right)+\left(m+1\right)d_{\text{p53}}P_{m+1,n}\left(t\right) \\ +\left(n+1\right)d_{\text{A}}P_{m,n+1}\left(t\right)-\left(g_{\text{p53}}^{(m)}P_{B}\left(B\geq1\right)+g_{\text{A}}+md_{\text{p53}}+nd_{\text{A}}\right)P_{m,n}\left(t\right) \end{matrix}$ (S25)

where $m<n$. And the probability density function of the FPT (or FPT distribution) can be formally expressed as

$f_{T}\left(t\right)=\sum_{m\geq0} d_{\text{A}}(m+1)P_{m,m+1}\left(t\right)+\sum_{n>m\geq0} g_{\text{p53}}^{\left(m\right)}P_{B}\left(B\geq n-m\right)P_{m,n}\left(t\right)$ (S26)

In what follows, we will consider only the case that there is no feedback and the transcription rates are constants, i.e., $g_{\text{p53}}^{\left(m\right)}\equiv g_{\text{p53}}$ and $g_{\text{A}}^{\left(n\right)}\equiv g_{\text{A}}$. To determine the first several moments of the FPT to a fluctuating threshold, we need the specific expression of PFT distribution $f_{T}\left(t\right)$.

# 3 FPT distribution and its statistics in four different fluctuating thresholds

For clarity, we will distinguish four cases of the absorbing domain to solve the FPT problem formulated above. These cases are schematized in [Fig. S3](#figs3). Based on the finite state projection approach, we construct a new process for pair  and on the finite state-space

(S27)

where the states represent the numbers of proteins and . Here we introduce two numerical cutoffs for the numbers of proteins and : for and for . Without loss of generality, assume that $C_{\text{p53}}=C_{\text{A}}=C$ with $C$ being a known positive integer. In addition, we introduce an operator that will be used to the calculation of matrix $\mathbf{M}$ defined later.

In order to give the expression of $\mathbf{M}$, we also introduce operators, denoted by ,$\left(i=1,2,\cdots,n\right)$, which acts on matrices with the operation rule being as follows: , where $\mathbf{M}^{\left(i\right)}$ is a matrix whose order is the same as $\mathbf{M}$ but some components are zero, e.g., if $\mathbf{M}=\left(a_{ij}\right)_{3\times3}$, we obtain , where $\left(b_{ij}\right)_{2\times2}=\left(a_{ij}\right)_{2\times2}$ and the other elements are equal to zero. Specially, we always keep if $i=0$, where $\mathbf{0}$ has same order with $\mathbf{M}$.

Owing to big differences in analysis and calculation, we will separately discuss the cases of four kinds of absorbing domains.

**Fig. S3** **Schematic diagram for the possible patterns of the absorbing domain of variable**  **to reach barrier** $A$: (A) ;

(B); (C)

(D).

In figure, circles represent points in the state space, lines represent the boundary of absorbing domain, and arrows represent the direction of threshold crossing.

## 3.1 The absorbing domain is

If the trajectory of protein pair and is considered in the domain $\left\{\left.\left(p53,A\right)\right|p53=p53\left(t\right)<A\left(t\right)=A\right\}$ (referring to [Fig. S3*A*](#figs3)), the random variable by the time at which protein counts reaches protein counts for the first time, is defined as

. (S28)

The absorbing domain in this case is given by

(S29)

And the corresponding finite state-space for birth-death process is defined as

If we write , then its finite state-space is

(S30)

The chemical master equation for $P_{m,n}\left(t\right)$ can thus be written in the form of [Eq. S25](#eqs25) with $\left(m,n\right)\in\Omega$. For convenience, we introduce the denotation

$$\mathbf{P}_{m,n}\left(t\right)=\left[\mathbf{P}_{*,1},\mathbf{P}_{*,2},\mathbf{P}_{*,3},\cdots,\mathbf{P}_{*,C}\right]^{T}=\left[\left[P_{01},0,\cdots,0\right],\left[P_{02},P_{12},0,\cdots,0\right],\cdots,\left[P_{0,C},P_{1,C},\cdots,P_{C-1,C}\right]\right]^{T}$$

where the denotation $\mathbf{P}_{*,k}=\left[P_{0,k},P_{1,k},\cdots,P_{k-1,k},0,\cdots,0\right]$, $k\in1,2,\cdots,\mathrm{C}$.

Based on the sub-matrix operator , we can easily determine the matrix $\mathbf{M}$ in the master equation $\partial_{t}\mathbf{P}_{m,n}\left(t\right)=\mathbf{MP}_{m,n}\left(t\right)$ or in [Eq. S25](#eqs25) with $\left(m,n\right)\in\Omega$. The form of the matrix $\mathbf{M}$ is

$\mathbf{M}=\left[\begin{matrix} \mathbf{D}_{1} & \mathbf{U}_{1} & & & & \\ \mathbf{L}_{1} & \mathbf{D}_{2} & \mathbf{U}_{2} & & & \\ & \mathbf{L}_{2} & \mathbf{D}_{3} & \mathbf{U}_{3} & & \\ & & \ddots& \ddots& \ddots& \\ & & & \mathbf{L}_{C-2} & \mathbf{D}_{C-1} & \mathbf{U}_{C-1} \\ & & & & \mathbf{L}_{C-1} & \mathbf{D}_{C} \end{matrix}\right]$ (S31)

where

Here $\mathbf{I}_{C}$is the $C\times C$ identity matrix. And the matrix $\mathbf{M}_{d_{\text{p53}}}$ is $\mathbf{M}_{d_{\text{p53}}}=d_{\text{p53}}\text{diag}\left(\left[1,2,\ldots,C-1\right],1\right)-d_{\text{p53}}\text{diag}\left(\left[0,1\ldots,C-1\right],0\right)$, where symbol $\text{diag}\left(\mathbf{v},k\right)$ represents that the elements of vector $\mathbf{v}$are placed on the $k\text{th}$diagonal, where $k=0$ corresponds to the main diagonal, $k>0$ to the upper principal diagonal, and $k<0$ to the lower principal diagonal. That is,

$\mathbf{M}_{d_{\text{p53}}}=\left[\begin{matrix} 0 & d_{\text{p53}} & & & & \\ & -d_{\text{p53}} & 2d_{\text{p53}} & & & \\ & & -2d_{\text{p53}} & 3d_{\text{p53}} & & \\ & & & \ddots& \ddots& \\ & & & & -\left(C-2\right)d_{\text{p53}} & \left(C-1\right)d_{\text{p53}} \\ & & & & & -\left(C-1\right)d_{\text{p53}} \end{matrix}\right]$. (S32)

Moreover, we have $\mathbf{M}_{g_{\text{p53}}}=g_{\text{p53}}\mathbf{M}_{\text{burst}}$, where $\mathbf{M}_{\text{burst}}=-P_{B\geq1}\mathbf{I}_{C}+\sum_{k=1}^{C-1} P_{B=k}\text{diag}\left(\mathbf{e}_{C-k}^{T},-k\right)$. In the case of feedback, implying that $g_{\text{p53}}$ depends on the molecule number ($m$) of protein $p53$ , $\mathbf{M}_{g_{\text{p53}}^{\left(m\right)}}=\mathbf{M}_{\text{burst}}\mathbf{G}$, where $\mathbf{G}=\text{diag}\left(\left[g_{\text{p53}}^{(0)},g_{\text{p53}}^{(1)}\ldots,g_{\text{p53}}^{(C-1)}\right]\text{,}0\right)$.

$\mathbf{M}_{g_{\text{p53}}}=\left[\begin{matrix} -g_{\text{p53}}^{\left(0\right)}P_{B\geq1} & & & & & \\ g_{\text{p53}}^{\left(0\right)}P_{B=1} & -g_{\text{p53}}^{\left(1\right)}P_{B\geq1} & & & & \\ g_{\text{p53}}^{\left(0\right)}P_{B=2} & g_{\text{p53}}^{\left(1\right)}P_{B=1} & -g_{\text{p53}}^{\left(2\right)}P_{B\geq1} & & & \\ \vdots& \vdots& \vdots& \ddots& & \\ g_{\text{p53}}^{\left(0\right)}P{}_{B=C-2} & g_{\text{p53}}^{\left(1\right)}P_{B=C-3} & g_{\text{p53}}^{\left(2\right)}P_{B=C-4} & \cdots& -g_{\text{p53}}^{\left(C-2\right)}P_{B\geq1} & \\ g_{\text{p53}}^{\left(0\right)}P_{B=C-1} & g_{\text{p53}}^{\left(1\right)}P_{B=C-2} & g_{\text{p53}}^{\left(2\right)}P_{B=C-3} & \cdots& g_{\text{p53}}^{\left(C-2\right)}P_{B=1} & -g_{\text{p53}}^{\left(C-1\right)}P_{B\geq1} \end{matrix}\right]$ (S33)

Given a numerical cutoff $\left(C\right)$, the FPT distribution can thus be determined by the following way. Apparently, the probability of a state reaches the absorption domain given by in the infinitesimal time interval $\left(t,t+dt\right)$, is the sum of the following two terms: the first one is the probability that and a jump of size or large occurs in the time interval $\left[t,t+dt\right)$, and the second one is the degradation probability that occurs in the time interval $\left[t,t+dt\right)$. Thus, the probability density function of the first passage time (FPT) that a pair of proteins $\left(p53,A\right)$ reach absorbing domain is given by

$\begin{matrix} f_{T}\left(t\right)=\sum_{m=0}^{C-1} \left(m+1\right)d_{\text{A}}P_{m,m+1}\left(t\right)+\sum_{n=1}^{C} \sum_{m=0}^{n-1} g_{\text{p53}}^{\left(m\right)}P_{B\geq n-m}P_{m,n}\left(t\right) \\ =\sum_{\begin{matrix} n=1 \\ m=n-1 \end{matrix}}^{C} nd_{\text{A}}P_{m,n}\left(t\right)+\sum_{n=1}^{C} \sum_{m=0}^{n-1} g_{\text{p53}}^{\left(m\right)}P_{B\geq n-m}P_{m,n}\left(t\right) \\ =\mathbf{W}_{d_{\text{A}}}^{T}\mathbf{P}_{m,n}\left(t\right)+\mathbf{W}_{g_{\text{p53}}}^{T}\mathbf{P}_{m,n}\left(t\right) \\ \equiv\mathbf{W}^{T}\mathbf{P}_{m,n}\left(t\right) \end{matrix}$ (S34)

where we denote the column vector $\mathbf{W}_{d_{\text{A}}}=\left(\mathbf{W}_{d_{\text{y}}}^{n}\right)_{C\times1}$ with $\mathbf{W}_{d_{\text{A}}}^{n}=nd_{\text{A}}\mathbf{1}_{n}$, $n=1,2\cdots,C$, and define a column vector of length $C$, $\mathbf{1}_{i}=\left(0,\cdots0,1,0,\cdots,0\right)^{T}$, in which the only $i$th element is equal to 1 and other elements are all zero. Similarly, the column vector $\mathbf{W}_{g_{\text{p53}}}$ can be rewritten as $\mathbf{W}_{g_{\text{p53}}}=\left(\mathbf{W}_{g_{\text{p53}}}^{n}\right)_{C\times1}$ with $\mathbf{W}_{g_{\text{p53}}}^{n}=\sum_{m=1}^{n-1} g_{\text{p53}}^{\left(m\right)}P_{B\geq n-m}\mathbf{1}_{m+1}$, $n=1,2\cdots,C$. Thus, the column vector $\mathbf{W}$can be expressed as

$\mathbf{W}=\mathbf{W}_{g_{\text{p53}}}+\mathbf{W}_{d_{\text{A}}}=\left[\mathbf{W}_{1}^{\text{T}},\mathbf{W}_{2}^{\text{T}},\cdots,\mathbf{W}_{C}^{\text{T}}\right]^{T}$ (S35)

where $\mathbf{W}_{n}=\mathbf{W}_{d_{\text{A}}}^{n}+\mathbf{W}_{g_{\text{p53}}}^{n}=nd_{\text{A}}\mathbf{1}_{n}+\sum_{m=0}^{n-1} g_{\text{p53}}^{\left(m\right)}P_{B\geq n-m}\mathbf{1}_{m+1}$, $n=1,2\cdots,C$. In brevity, the involved vectors can be expressed as

$$\mathbf{W}_{d_{\text{A}}}^{T}=\left[\left[d_{\text{A}},0,\cdots,0\right],\left[0,2d_{\text{A}},0,\cdots,0\right],\cdots,\left[0,0,\cdots,0,Cd_{\text{A}}\right]\right],$$

$$\mathbf{W}_{g_{\text{p53}}}^{T}=\left[\left[g_{\text{p53}}^{\left(0\right)}P_{B\geq1},0,\cdots,0\right],\left[g_{\text{p53}}^{\left(0\right)}P_{B\geq2},g_{\text{p53}}^{\left(1\right)}P_{B\geq1},0,\cdots,0\right],\cdots,\left[g_{\text{p53}}^{\left(0\right)}P_{B\geq C},g_{\text{p53}}^{\left(1\right)}P_{B\geq C-1},\cdots,g_{\text{p53}}^{\left(C-1\right)}P_{B\geq1}\right]\right],$$

$$\mathbf{W}^{T}=\left[\left[g_{\text{p53}}^{\left(0\right)}P_{B\geq1}+d_{\text{A}},0,\cdots,0\right],\left[g_{\text{p53}}^{\left(0\right)}P_{B\geq2},g_{\text{p53}}^{\left(1\right)}P_{B\geq1}+2d_{\text{A}},0,\cdots,0\right],\cdots,\left[g_{\text{p53}}^{\left(0\right)}P_{B\geq C},g_{\text{p53}}^{\left(1\right)}P_{B\geq C-1},\cdots,g_{\text{p53}}^{\left(C-1\right)}P_{B\geq1}+Cd_{\text{A}}\right]\right]$$

The formulation for calculating moments of the FPT is the same as [Eq. S17](#eqs17).

Numerical results for mean FPT and timing variability are shown in [Figs. S4](#figs4) and [S5](#figs5), which they are the same as [Figs. 3](#figs4) and 6 in main file.

**Fig. S4 Comparison between the effects of fixed and fluctuating thresholds on timing**. (A) Timing mean as a function of event threshold in two different kinds of fixed and fluctuating threshold, where the inset shows the timing mean as a function of event threshold on the logarithmic scale. (B) A different demonstration of the results in (A), showing the difference of timing mean in the case of fixed threshold minus that in the case of fluctuating threshold, where two insets show FPT distributions for two different event thresholds (indicated by empty circle and triangle) corresponding to $A_{\text{threshold}}=4$ and $A_{\text{threshold}}=13$. (C) Timing variability as a function of event threshold in two different kinds of thresholds, where the empty circle is the crossing point of two curves, and stars represent the event threshold that makes timing variability reach the minimum. (D) As a supplement of (C), the difference of timing variability in the case of fixed threshold minus that in the case of fluctuating threshold, where the inset shows the critical threshold ($\text{RP}$) as a function of transcription rate ($g_{\text{p53}}$). In (A) and (C), the parameter values are set as $g_{\text{p53}}=5$, $d_{\text{p53}}=1$, $b=1$, $p53_{\max}=30$,$d_{\text{A}}=1$, and $g_{\text{A}}=1\sim15$. Here we always keep $d_{\text{A}}=1$, thus the event threshold is decided by $g_{\text{A}}$, i.e.,$\text{event threshold}=g_{\text{A}}=1\sim15$. That is, if the fixed threshold is $A_{\text{threshold}}=10$, then the fluctuating threshold corresponds to $g_{\text{A}}=10$ and $d_{\text{A}}=1$. The inset in (D) corresponds to$d_{\text{p53}}=1$, $b=1$, $A_{\text{threshold}}=g_{\text{A}}=1\sim10$, $d_{\text{A}}=1$, $g_{\text{p53}}=1\sim10$, and $p53_{\max}=30$.

**Fig. S5** **Effect of mean burst sizes on event timing**. (A)(B) Timing mean and timing variability as a function of mean burst size ($b$) in two cases of fixed (blue curve) and fluctuating (red curve) threshold, where the inset shows a partial enlarged diagram, and $RP$ represents a critical point for reversing. (C)(D) Heatmap respectively showing timing mean and timing variability as a function of both event threshold and $b$, where the white curves are contour lines, and the red curve consists of the minimal timing variability in the case of a given event threshold, while the white stair-like line is minimal that in the case of a fixed mean burst size, similar to the star in Fig. 3(C) in the main file. In (A)-(D), the parameter values are set as $g_{\text{p53}}=5$, $d_{\text{p53}}=1$, $p53_{\max}=30$. In (A) and (B), the threshold is set as $A_{\text{thresold}}=g_{\text{A}}/d_{\text{A}}$, with $g_{\text{A}}=10$ and $d_{\text{A}}=1$, and. whereas in (C) and (D), $d_{\text{A}}=1$ and $g_{\text{A}}$ is changed in $A_{\text{thresold}}=g_{\text{A}}/d_{\text{A}}$.

## 3.2 The absorbing domain is

If the trajectory $p53,A()$ of protein pair and is considered in two domains and (referring to [Fig. S3*B*](#figs3)), we define the random variable by the time at which protein counts reach protein counts for the first time, that is,

(S36)

The absorbing domain in this case is given by

. (S37)

The corresponding finite state-space for birth-death process is defined as , where

Then, we easily write the finite state-space , which consists of two parts , i.e., , where we denote respectively

(S38)

(S39)

The corresponding chemical master equation for $P_{m,n}\left(t\right)$ satisfies the form of [Eq. S25](#eqs25) with $\left(m,n\right)\in\Omega$. Denote

$$\begin{matrix} \mathbf{P}_{m,n}\left(t\right)=\left[P_{*,0},P_{*,1},\cdots,P_{*,x_{C}-1},P_{*,x_{C}},P_{*,x_{C}+1},\cdots,P_{*,C}\right]^{T} \\ =\left[\left[P_{0,0},P_{1,0},\cdots,P_{x_{C}-1,0},0,\cdots,0\right],\cdots,\right.\left[P_{0,x_{C}-1},P_{1,x_{C}-1},\cdots,P_{x_{C}-1,x_{C}-1},0,\cdots,0\right], \\ \left[P_{0,x_{C}},P_{1,x_{C}},\cdots,P_{x_{C}-1,x_{C}},0,\cdots,0\right],\left[P_{0,x_{C}+1},\cdots,P_{x_{C}-1,x_{C}+1},P_{x_{C},x_{C}+1},0,\cdots,0\right], \\ \left.\cdots,\left[P_{0,C},P_{1,C},\cdots,P_{C-1,C}\right]\right]^{T} \end{matrix}$$

Based on the sub-matrix operator , we can easily determine matrix $\mathbf{M}$ in the master equation $\partial_{t}\mathbf{P}_{m,n}\left(t\right)=\mathbf{MP}_{m,n}\left(t\right)$ or in [Eq. S25](#eqs25) with $\left(m,n\right)\in\Omega$. The form of the matrix $\mathbf{M}$ is

$\mathbf{M}=\left[\begin{matrix} \mathbf{D}_{0} & \mathbf{U}_{0} & & & & \\ \mathbf{L}_{0} & \mathbf{D}_{1} & \mathbf{U}_{1} & & & \\ & \mathbf{L}_{1} & \mathbf{D}_{2} & \mathbf{U}_{2} & & \\ & & \ddots& \ddots& \ddots& \\ & & & \mathbf{L}_{C-2} & \mathbf{D}_{C-1} & \mathbf{U}_{C-1} \\ & & & & \mathbf{L}_{C-1} & \mathbf{D}_{C} \end{matrix}\right]$ (S40)

where,

Here, $\mathbf{I}_{C}$, $\mathbf{M}_{d_{\text{p53}}}$and $\mathbf{M}_{g_{\text{p53}}}$ have the same definition as the case with absorbing domain .

Note that the probability that a state reaches the absorbing domain defined by in the infinitesimal time interval $\left(t,t+dt\right)$ is the sum of the following three terms: the probability that and a jump of size or large occurs in the time interval $\left[t,t+dt\right)$, the degradation probability that occurs in the time interval $\left[t,t+dt\right)$, and the probability that and a jump of size or large occurs in the time interval $\left[t,t+dt\right)$. Thus, the probability density function of the FPT that $p53\left(t\right),A\left(t\right)()$ reaches absorbing domain is given by

$\begin{matrix} f_{T}\left(t\right)=\sum_{m=x_{C}}^{C-1} \left(m+1\right)d_{\text{A}}P_{m,m+1}\left(t\right)+\sum_{n=x_{C}}^{C} \sum_{m=0}^{n-1} g_{\text{p53}}^{\left(m\right)}P_{B\geq n-m}P_{m,n}\left(t\right)+\sum_{n=0}^{x_{C}-1} \sum_{m=0}^{x_{C}-1} g_{\text{p53}}^{\left(m\right)}P_{B\geq x_{C}-m}P_{m,n}\left(t\right) \\ =\sum_{\begin{matrix} n=x_{C}+1 \\ m=n-1 \end{matrix}}^{C} nd_{\text{A}}P_{m,n}\left(t\right)+\sum_{n=x_{C}}^{C} \sum_{m=0}^{n-1} g_{\text{p53}}^{\left(m\right)}P_{B\geq n-m}P_{m,n}\left(t\right)+\sum_{n=0}^{x_{C}-1} \sum_{m=0}^{x_{C}-1} g_{\text{p53}}^{\left(m\right)}P_{B\geq x_{C}-m}P_{m,n}\left(t\right) \\ =W_{d_{\text{A}}}^{T}\mathbf{P}_{m,n}\left(t\right)+W_{g_{\text{p53}}}^{\left(1\right)T}\mathbf{P}_{m,n}\left(t\right)+W_{g_{\text{p53}}}^{\left(2\right)T}\mathbf{P}_{m,n}\left(t\right) \\ \equiv W^{T}\mathbf{P}_{m,n}\left(t\right) \end{matrix}$ (S41)

where we denote respectively three column vectors

$$\left\{\begin{matrix} W_{d_{\text{A}}}=\left(W_{d_{\text{A}}}^{n}\right)_{\left(C+1\right)\times1} \\ W_{g_{\text{p53}}}^{\left(1\right)}=\left(W_{g_{\text{p53}}}^{n}\right)_{\left(C+1\right)\times1} \\ W_{g_{\text{p53}}}^{\left(2\right)}=\left(\hat{W}_{g_{\text{p53}}}^{n}\right)_{\left(C+1\right)\times1} \end{matrix}\right.$$

which the corresponding vector is given by

$$\mathbf{W}_{d_{\text{A}}}^{n}=\left\{\begin{matrix} \mathbf{0},\text{ }n=0,1,\cdots,x_{C}; \\ nd_{\text{A}}\mathbf{1}_{n},\text{ }n=x_{C}+1,x_{C}+2,\cdots,C. \end{matrix}\right.$$

$$\mathbf{W}_{g_{\text{p53}}}^{n}=\left\{\begin{matrix} \mathbf{0},\text{ }n=0,1,\cdots,x_{C}-1;\text{ } \\ \sum_{m=0}^{n-1} g_{\text{p53}}^{\left(m\right)}P_{B\geq n-m}\mathbf{1}_{m+1},\text{ }n=x_{C},x_{C}+1,\cdots,C. \end{matrix}\right.$$

$$\hat{\mathbf{W}}_{g_{\text{p53}}}^{n}=\left\{\begin{matrix} \text{ }\sum_{m=0}^{x_{C}-1} g_{\text{p53}}^{\left(m\right)}P_{B\geq x_{C}-m}\mathbf{1}_{m+1},\text{ }\text{ }n=0,1,\cdots,x_{C}-1; \\ \mathbf{0},\text{ }n=x_{C},x_{C}+1,\cdots,C. \end{matrix}\right.$$

Thus, the column vector $\mathbf{W}$ takes the following form

$\mathbf{W}=\mathbf{W}_{g_{\text{p53}}}^{\left(1\right)}+\mathbf{W}_{g_{\text{p53}}}^{\left(2\right)}+\mathbf{W}_{d_{\text{A}}}=\left[\mathbf{W}_{0}^{T},\mathbf{W}_{1}^{T},\mathbf{W}_{2}^{T},\cdots,\mathbf{W}_{n}^{T}\right]^{T}$ (S42)

where $\mathbf{W}_{n}=\mathbf{W}_{g_{\text{p53}}}^{n}+\hat{\mathbf{W}}_{g_{\text{p53}}}^{n}+\mathbf{W}_{d_{\text{A}}}^{n}$, which can be expressed as

$$\mathbf{W}_{n}=\left\{\begin{matrix} \text{ }\sum_{m=0}^{x_{C}-1} g_{\text{p53}}^{\left(m\right)}P_{B\geq x_{C}-m}\mathbf{1}_{m+1},\text{ }\text{ }n=0,1,\cdots,x_{C}-1,x_{C}; \\ nd_{\text{A}}\mathbf{1}_{n}+\sum_{m=0}^{n-1} g_{\text{p53}}^{\left(m\right)}P_{B\geq n-m}\mathbf{1}_{m+1},\text{ }n=x_{C}+1,x_{C}+2\cdots,C. \end{matrix}\right.$$

In brevity, the involved vectors can be expressed as

$$W_{d_{\text{A}}}^{T}=\left[\left[0,\cdots,0\right],\cdots,\left[0,\cdots,0\right],\left[0,\cdots,0,\left(x_{C}+1\right)d_{\text{A}},0,\cdots,0\right],\right.\left.\left[0,\cdots,0,\left(x_{C}+2\right)d_{\text{A}},0,\cdots,0\right],\cdots,\left[0,\cdots,0,Cd_{\text{A}}\right]\right]$$

$$\begin{matrix} W_{g_{\text{p53}}}^{\left(1\right)T}=\left[\left[0,\cdots,0\right],\cdots,\left[0,\cdots,0\right],\left[g_{\text{p53}}^{\left(0\right)}P_{B\geq x_{C}},g_{\text{p53}}^{\left(1\right)}P_{B\geq x_{C}-1},\cdots,g_{\text{p53}}^{\left(x_{C}-1\right)}P_{B\geq1},0,\cdots,0\right],\right. \\ \left[g_{\text{p53}}^{\left(0\right)}P_{B\geq x_{C}+1},g_{\text{p53}}^{\left(1\right)}P_{B\geq x_{C}},\cdots,g_{\text{p53}}^{\left(x_{C}\right)}P_{B\geq1},0,\cdots,0\right],\left.\cdots,\left[g_{\text{p53}}^{\left(0\right)}P_{B\geq C},g_{\text{p53}}^{\left(1\right)}P_{B\geq C-1},\cdots,g_{\text{p53}}^{\left(C-1\right)}P_{B\geq1}\right]\right] \end{matrix}$$

$$\begin{matrix} W_{g_{\text{p53}}}^{\left(2\right)T}=\left[\left[g_{\text{p53}}^{\left(0\right)}P_{B\geq x_{C}},g_{\text{p53}}^{\left(1\right)}P_{B\geq x_{C}-1},\cdots,g_{\text{p53}}^{\left(x_{C}-1\right)}P_{B\geq1},0,\cdots,0\right],\right.\left[g_{\text{p53}}^{\left(0\right)}P_{B\geq x_{C}},g_{\text{p53}}^{\left(1\right)}P_{B\geq x_{C}-1},\cdots,g_{\text{p53}}^{\left(x_{C}-1\right)}P_{B\geq1},0,\cdots,0\right],\cdots, \\ \left.\left[g_{\text{p53}}^{\left(0\right)}P_{B\geq x_{C}},g_{\text{p53}}^{\left(1\right)}P_{B\geq x_{C}-1},\cdots,g_{\text{p53}}^{\left(x_{C}-1\right)}P_{B\geq1},0,\cdots,0\right],\left[0,\cdots,0\right],\cdots,\left[0,\cdots,0\right]\right] \end{matrix}$$

$$\begin{matrix} W^{T}=\left[\left[g_{\text{p53}}^{\left(0\right)}P_{B\geq x_{C}},g_{\text{p53}}^{\left(1\right)}P_{B\geq x_{C}-1},\cdots,g_{\text{p53}}^{\left(x_{C}-1\right)}P_{B\geq1},0,\cdots,0\right],\cdots,\right.\left[g_{\text{p53}}^{\left(0\right)}P_{B\geq x_{C}},g_{\text{p53}}^{\left(1\right)}P_{B\geq x_{C}-1},\cdots,g_{\text{p53}}^{\left(x_{C}-1\right)}P_{B\geq1},0,\cdots,0\right], \\ \left[g_{\text{p53}}^{\left(0\right)}P_{B\geq x_{C}+1},g_{\text{p53}}^{\left(1\right)}P_{B\geq x_{C}},\cdots,\left(x_{C}+1\right)d_{\text{A}}+g_{\text{p53}}^{\left(x_{C}\right)}P_{B\geq1},0,\cdots,0\right],\cdots,\left.\left[g_{\text{p53}}^{\left(0\right)}P_{B\geq C},g_{\text{p53}}^{\left(1\right)}P_{B\geq C-1},\cdots,Cd_{\text{A}}+g_{\text{p53}}^{\left(C-1\right)}P_{B\geq1}\right]\right]. \end{matrix}$$

The formulation for calculating the moments of the FPT is the same as [Eq. S17](#eqs17).

Numerical results for mean FPT and timing variability are shown in [Fig. S6](#figs6).

**Fig.S6 Characteristic of the curve for timing mean or timing variability vs event threshold in the case of absorbing domain** . (A) Schematic for PFT when an absorbing domain is defined by , where arrows represent the direction of threshold crossing. (B) Timing mean as a function of event threshold for two different kinds of thresholds, where empty circle represents the crossing point $P$ of two curves for the mean FPT in two cases of event threshold. (C) Timing variability as a function of event threshold for two different kinds of thresholds, where two empty circles are the crossing points of two curves, denoted by $RP_{1}$ and $RP_{2}$. In (B) and (C), the parameter values are set as $x_{\text{C}}=5$, $g_{\text{p53}}=5$, $d_{\text{p53}}=1$, $b=1$, the cutoff constant is set as $p53_{\max}=50$. The threshold is set as $A_{\text{thresold}}=g_{\text{A}}/d_{\text{A}}$, where keeps always $d_{\text{A}}=1$. If the fixed threshold is set as $A_{\text{threshold}}=10$, the fluctuating threshold corresponds to $g_{\text{A}}=10$ and $d_{\text{A}}=1$.

## 3.3 The absorbing domain is

If the trajectory $p53,A()$ of protein pair and is considered in two domain and (referring to [Fig. S3*C*](#figs3)), we define the random variable as the time that protein counts reach protein counts for the first time, that is,

$T=\min\left\{t:\left.p53\left(t\right)\geq A\left(t\right),A\left(t\right)\geq y_{C}\right|p53\left(0\right)=m<A\left(0\right)=n\right\}$ (S43)

The absorbing domain in this case is given by

. (S44)

The corresponding finite state-space for birth-death process with , where

, .

Then we easily write its finite state-space , which consists of two parts, i.e., , where we denote respectively

, (S45)

. (S46)

The corresponding chemical master equation for $P_{m,n}\left(t\right)$ takes the form of [Eq.S25](#eqs25) with $\left(m,n\right)\in\Omega$. Denote

$$\begin{matrix} P_{m,n}\left(t\right)=\left[P_{*,0},P_{*,1},\cdots,P_{*,y_{C}-1},P_{*,y_{C}},P_{*,y_{C}+1},\cdots,P_{*,C}\right]^{T} \\ =\left[\left[P_{0,0},P_{1,0},\cdots,P_{C-1,0}\right],\cdots,\left[P_{0,y_{C}-1},P_{1,y_{C}-1},\cdots,P_{C-1,y_{C}-1}\right],\left[P_{0,y_{C}},P_{1,y_{C}},\cdots,P_{y_{C}-1,y_{C}},0,\cdots,0\right],\right. \\ \left.\left[P_{0,y_{C}+1},P_{1,y_{C}+1},\cdots,P_{y_{C},y_{C}+1},0,\cdots,0\right],\cdots,\left[P_{0,C},P_{1,C},\cdots,P_{C-1,C}\right]\right]^{T} \end{matrix}$$

Note that here $P_{*,k}$ has two forms,

$$\left\{\begin{matrix} P_{*,\mathrm{k}}=\left[P_{0,k},P_{1,k},\cdots,P_{C-1,k}\right],\text{ }k=0,1,\cdots,y_{C}-1; \\ P_{*,\mathrm{k}}=\left[P_{0,k},P_{1,k},\cdots,P_{k-1,k},0,\cdots,0\right],\text{ }k=y_{C},y_{C}+1,\cdots,C. \end{matrix}\right.$$

It is obvious that the matrix $\mathbf{M}$, which satisfies the master equation $\partial_{t}\mathbf{P}_{m,n}\left(t\right)=\mathbf{MP}_{m,n}\left(t\right)$ or in [Eq. S25](#eqs25) with $\left(m,n\right)\in\Omega$, is also easily determined for the sub-matrix operator in this case, i.e.,

$\mathbf{M}=\left[\begin{matrix} \mathbf{D}_{0} & \mathbf{U}_{0} & & & & \\ \mathbf{L}_{0} & \mathbf{D}_{1} & \mathbf{U}_{1} & & & \\ & \mathbf{L}_{1} & \mathbf{D}_{2} & \mathbf{U}_{2} & & \\ & & \ddots& \ddots& \ddots& \\ & & & \mathbf{L}_{C-2} & \mathbf{D}_{C-1} & \mathbf{U}_{C-1} \\ & & & & \mathbf{L}_{C-1} & \mathbf{D}_{C} \end{matrix}\right]$ (S47)

where,

Here, $\mathbf{I}_{C}$, $\mathbf{M}_{d_{\text{p53}}}$and $\mathbf{M}_{g_{\text{p53}}}$ have same definition with above case with absorbing domain .

Similarly, given a numerical cutoff $\left(C\right)$, we can determine the probability that a state reaches the absorbing domain defined by in the infinitesimal time interval $\left(t,t+dt\right)$ in this case. This probability is the sum of the following three terms: the probability that and a jump of size or larger occurs in the time interval $\left[t,t+dt\right)$, the degradation probability that occurs in the time interval $\left[t,t+dt\right)$, and the transcription probability that with occurs in the time interval $\left[t,t+dt\right)$. Thus, the probability density function of the FPT that $\left(p53,A\right)$ reach absorbing domain is given by

(S48)

where we denote respectively three column vectors

$$\left\{\begin{matrix} W_{d_{\text{A}}}=\left(W_{d_{\text{A}}}^{n}\right)_{\left(C+1\right)\times1} \\ W_{g_{\text{p53}}}=\left(W_{g_{\text{p53}}}^{n}\right)_{\left(C+1\right)\times1} \\ W_{g_{\text{A}}}=\left(W_{g_{\text{A}}}^{n}\right)_{\left(C+1\right)\times1} \end{matrix}\right.$$

with

$$\mathbf{W}_{d_{\text{A}}}^{n}=\left\{\begin{matrix} \mathbf{0},\text{ }n=0,1,\cdots,y_{C}; \\ nd_{\text{A}}\mathbf{1}_{n},\text{ }n=y_{C}+1,y_{C}+2\cdots,C. \end{matrix}\right.$$

$$\mathbf{W}_{g_{\text{p53}}}^{n}=\left\{\begin{matrix} \mathbf{0},\text{ }n=0,1,\cdots,y_{C}-1;\text{ } \\ \sum_{m=0}^{n-1} g_{\text{p53}}^{\left(m\right)}P_{B\geq n-m}\mathbf{1}_{m+1},\text{ }n=y_{C},y_{C}+1\cdots,C. \end{matrix}\right.$$

$$\mathbf{W}_{g_{\text{A}}}^{n}=\left\{\begin{matrix} \text{ }\sum_{m=y_{C}}^{C-1} g_{A}\mathbf{1}_{m+1},\text{ }\text{ }n=y_{C}-1; \\ \mathbf{0},\text{ }n\neq y_{C}-1. \end{matrix}\right.$$

Thus, the column vector $\mathbf{W}$ can be defined as the following form

$\mathbf{W}=\mathbf{W}_{g_{\text{p53}}}+\mathbf{W}_{g_{\text{A}}}+\mathbf{W}_{d_{\text{A}}}=\left[\mathbf{W}_{0}^{T},\mathbf{W}_{1}^{T},\mathbf{W}_{2}^{T},\cdots,\mathbf{W}_{n}^{T}\right]^{T}$ (S49)

where $\mathbf{W}_{n}=\mathbf{W}_{g_{\text{p53}}}^{n}+\mathbf{W}_{g_{\text{A}}}^{n}+\mathbf{W}_{d_{\text{A}}}^{n}$, which can be expressed as

$$\mathbf{W}_{n}=\left\{\begin{matrix} \mathbf{0},\text{ }n=0,1,\cdots,y_{C}-2; \\ \text{ }\sum_{m=y_{C}}^{C-1} g_{A}\mathbf{1}_{m+1},\text{ }\text{ }n=y_{C}-1; \\ \sum_{m=0}^{n-1} g_{\text{p53}}^{\left(m\right)}P_{B\geq n-m}\mathbf{1}_{m+1},\text{ }n=y_{C}; \\ nd_{\text{A}}\mathbf{1}_{n}+\sum_{m=0}^{n-1} g_{\text{p53}}^{\left(m\right)}P_{B\geq n-m}\mathbf{1}_{m+1},\text{ }n=y_{C}+1,y_{C}+2\cdots,C. \end{matrix}\right.$$

In brevity, the involved vectors can be expressed as

$$W_{d_{\text{A}}}^{T}=\left[\left[0,\cdots,0\right],\cdots,\left[0,\cdots,0\right],\left[0,\cdots,0,\left(y_{C}+1\right)d_{\text{A}},0,\cdots,0\right],\left[0,\cdots,0,\left(y_{C}+2\right)d_{\text{A}},0,\cdots,0\right],\cdots,\left[0,\cdots,0,Cd_{\text{A}}\right]\right]$$

$$W_{g_{\text{A}}}^{T}=\left[\left[0,\cdots,0\right],\cdots,\left[0,\cdots,0\right],\left[0,\cdots,0,g_{\text{A}},g_{\text{A}},\cdots,g_{\text{A}}\right],\left[0,\cdots,0\right],\cdots,\left[0,\cdots,0\right]\right]$$

The formulation for calculating the moments of the FPT is the same as [Eq. S17](#eqs17).

Numerical results for mean FPT and timing variability are shown in [Fig. S7](#figs7).

**Fig. S7 Characteristic of the curve for timing mean or timing variability vs event threshold in the case of absorbing domain** . (A) Schematic for PFT when an absorbing domain is defined by . (B) Timing mean as a function of event threshold for two different kinds of thresholds, where there is a crossing point $P$ which is the same mean with the [Fig. S6*B*](#figs6). (C) Timing variability as a function of event threshold for two different kinds of thresholds, where two empty circle represent the crossing point of two curves, denoted by $RP_{1}$ and $RP_{2}$. In (B) and (C), the parameter values are set as $g_{\text{p53}}=5$, $d_{\text{p53}}=1$, $b=1$, $y_{\text{C}}=5$, and $p53_{\max}=50$. The threshold is set as $A_{\text{thresold}}=g_{\text{A}}/d_{\text{A}}$, where keeps always $d_{\text{A}}=1$, thus $A_{\text{thresold}}=1\sim15$ is decided by changing $g_{A}$. If the fixed threshold is set as $A_{\text{threshold}}=10$, the fluctuating threshold corresponds to $g_{\text{A}}=10$ and $d_{\text{A}}=1$.

## 3.4 The absorbing domain is

Here, we analyze the case of absorbing domain $\left\{\left.(p53,A)\right|p53\left(t\right)\geq A\left(t\right),\text{ }p53\left(t\right)\geq x_{C},A\left(t\right)\geq y_{C}\right\}$. Obviously, it becomes the above case **S3.1** when $x_{C}=y_{C}=0$ (referring to [Fig. S3*A*](#figs3)), and the above case **S3.2** when $x_{C}>0,y_{C}=0$ (referring to [Fig. S3*B*](#figs3)), and the above case **S3.3** if $x_{C}=y_{C}>0$ or$y_{C}>x_{C}\geq0$ (referring to [Fig. S3*C*](#figs3)). However, the case of $x_{C}>y_{C}>0$is an exception since it does not belong to these three cases. This case will be discussed in **S3.4** of this section wherein the absorbing domain is specified as with $x_{C}>y_{C}>0$.

If the trajectory $p53,A()$ of protein pair and is considered in three domains (referring to [Fig. S3*D*](#figs3)): , , and , we define the random variable by the time at which protein counts reach protein counts for the first time, that is,

$T=\min\left\{t:\left.p53\left(t\right)\geq A\left(t\right),p53\left(t\right)\geq x_{C},A\left(t\right)\geq y_{C}\right|p53\left(0\right)=m<A\left(0\right)=n\right\}$ (S50)

The absorbing domain in this case is given by

(S51)

And the corresponding finite state-space for birth-death process is given by , where

,

,

.

Then we easily write the finite state-space , which consists of three parts , i.e., , where we denote respectively

, (S52)

, (S53)

. (S54)

Moreover, the corresponding chemical master equation for $P_{m,n}\left(t\right)$ takes the form of [Eq. S25](#eqs25) with $\left(m,n\right)\in\Omega$ , where we denote

$$\begin{matrix} P_{m,n}\left(t\right)=\left[P_{*,0},\cdots,P_{*,y_{C}-1},P_{*,y_{C}},P_{*,y_{C}+1},\cdots,P_{*,x_{C}},P_{*,x_{C}+1},\cdots,P_{*,C}\right]^{T} \\ =\left[\left[P_{0,0},P_{1,0},\cdots,P_{C-1,0}\right],\cdots,\left[P_{0,y_{C}-1},P_{1,y_{C}-1},\cdots,P_{C-1,y_{C}-1}\right],\right.\left[P_{0,y_{C}},P_{1,y_{C}},\cdots,P_{x_{C}-1,y_{C}},0,\cdots,0\right], \\ \left[P_{0,y_{C}+1},P_{1,y_{C}+1},\cdots,P_{x_{C}-1,y_{C}+1},0,\cdots,0\right],\cdots,\left[P_{0,x_{C}},P_{1,x_{C}},\cdots,P_{x_{C}-1,x_{C}},0,\cdots,0\right], \\ \left[P_{0,x_{C}+1},P_{1,x_{C}+1},\cdots,P_{x_{C},x_{C}+1},0,\cdots,0\right],\left.\cdots,\left[P_{0,\mathrm{C}},P_{1,\mathrm{C}},\cdots,P_{C-1,\mathrm{C}}\right]\right]^{T} \end{matrix}$$

Note that $x_{C}>y_{C}$ and $P_{*,k}$ has three forms,

$$\left\{\begin{matrix} P_{*,\mathrm{k}}=\left[P_{0,k},P_{1,k},\cdots,P_{C-1,k}\right],\text{ }k=0,1,\cdots,y_{C}-1; \\ P_{*,\mathrm{k}}=\left[P_{0,k},P_{1,k},\cdots,P_{x_{C}-1,k},0,\cdots,0\right],\text{ }k=y_{C},y_{C}+1,\cdots,x_{C}-1. \\ P_{*,\mathrm{k}}=\left[P_{0,k},P_{1,k},\cdots,P_{k-1,k},0,\cdots,0\right],\text{ }k=x_{C},x_{C}+1,\cdots,C. \end{matrix}\right.$$

Similarity, we easily determine the matrix $\mathbf{M}$ in equation $\partial_{t}\mathbf{P}_{m,n}\left(t\right)=\mathbf{MP}_{m,n}\left(t\right)$with $\left(m,n\right)\in\Omega$for the sub-matrix operator , that is

$\mathbf{M}=\left[\begin{matrix} \mathbf{D}_{0} & \mathbf{U}_{0} & & & & \\ \mathbf{L}_{0} & \mathbf{D}_{1} & \mathbf{U}_{1} & & & \\ & \mathbf{L}_{1} & \mathbf{D}_{2} & \mathbf{U}_{2} & & \\ & & \ddots& \ddots& \ddots& \\ & & & \mathbf{L}_{C-2} & \mathbf{D}_{C-1} & \mathbf{U}_{C-1} \\ & & & & \mathbf{L}_{C-1} & \mathbf{D}_{C} \end{matrix}\right]$ (S55)

where

Here, $\mathbf{I}_{C}$, $\mathbf{M}_{d_{\text{p53}}}$and $\mathbf{M}_{g_{\text{p53}}}$ have same definition with above case.

In this case, for the given numerical cutoff $\left(C\right)$, we can determine the probability that a state reaches the absorbing domain defined by with $x_{C}>y_{C}$ in the infinitesimal time interval $\left(t,t+dt\right)$ . This probability is the sum of the following four terms: the probability that and a jump of sizeor large occurs in the time interval $\left[t,t+dt\right)$. the probability that and a jump of sizeor large occurs in the time interval $\left[t,t+dt\right)$, the degradation probability that with occurs in the time interval $\left[t,t+dt\right)$, and the transcription probability that with occurs in the time interval $\left[t,t+dt\right)$. Thus, the probability density function of the FPT that $\left(p53,A\right)$ reach absorbing domain is given by

(S56)

where we denote respectively three column vectors

$$\left\{\begin{matrix} W_{d_{\text{A}}}=\left(W_{d_{\text{A}}}^{n}\right)_{\left(C+1\right)\times1} \\ W_{g_{\text{p53}}}^{\left(1\right)}=\left(W_{g_{\text{p53}}}^{n}\right)_{\left(C+1\right)\times1} \\ W_{g_{\text{p53}}}^{\left(2\right)}=\left(\hat{W}_{g_{\text{p53}}}^{n}\right)_{\left(C+1\right)\times1} \\ W_{g_{\text{A}}}=\left(W_{g_{\text{A}}}^{n}\right)_{\left(C+1\right)\times1} \end{matrix}\right.$$

with

$$\mathbf{W}_{d_{\text{A}}}^{n}=\left\{\begin{matrix} \mathbf{0},\text{ }n=0,1,\cdots,x_{C}; \\ nd_{\text{A}}\mathbf{1}_{n},\text{ }n=x_{C}+1,x_{C}+2\cdots,C. \end{matrix}\right.$$

$$\mathbf{W}_{g_{\text{p53}}}^{n}=\left\{\begin{matrix} \mathbf{0},\text{ }n=0,1,\cdots,x_{C}-1;\text{ } \\ \sum_{m=0}^{n-1} g_{\text{p53}}^{\left(m\right)}P_{B\geq n-m}\mathbf{1}_{m+1},\text{ }n=x_{C},x_{C}+1,\cdots,C. \end{matrix}\right.$$

$$\hat{\mathbf{W}}_{g_{\text{p53}}}^{n}=\left\{\begin{matrix} \mathbf{0},\text{ }n=0,1,\cdots,y_{C}-1,x_{C},x_{C}+1,\cdots,C; \\ \sum_{m=0}^{x_{C}-1} g_{\text{p53}}^{\left(m\right)}P_{B\geq x_{C}-m}\mathbf{1}_{m+1},\text{ }n=y_{C},y_{C}+1,\cdots,x_{C}-1. \end{matrix}\right.$$

$$\mathbf{W}_{g_{\text{A}}}^{n}=\left\{\begin{matrix} \text{ }\sum_{m=y_{C}}^{C-1} g_{A}\mathbf{1}_{m+1},\text{ }\text{ }n=y_{C}-1; \\ \mathbf{0},\text{ }n\neq y_{C}-1. \end{matrix}\right.$$

Thus, the column vector $\mathbf{W}$ can be expressed as the follow form

$\mathbf{W}=\mathbf{W}_{g_{\text{p53}}}^{\left(1\right)}+\mathbf{W}_{g_{\text{p53}}}^{\left(2\right)}+\mathbf{W}_{g_{\text{A}}}+\mathbf{W}_{d_{\text{A}}}=\left[\mathbf{W}_{0}^{T},\mathbf{W}_{1}^{T},\mathbf{W}_{2}^{T},\cdots,\mathbf{W}_{n}^{T}\right]^{T}$ (S57)

where $\mathbf{W}_{n}=\mathbf{W}_{g_{\text{p53}}}^{n}+\hat{\mathbf{W}}_{g_{\text{p53}}}^{n}+\mathbf{W}_{g_{\text{A}}}^{n}+\mathbf{W}_{d_{\text{A}}}^{n}$, which can be expressed as

$$\mathbf{W}_{n}=\left\{\begin{matrix} \mathbf{0},\text{ }n=0,1,\cdots,y_{C}-2; \\ \text{ }\sum_{m=x_{C}}^{C-1} g_{A}\mathbf{1}_{m+1},\text{ }\text{ }n=y_{C}-1; \\ \sum_{m=0}^{x_{C}-1} g_{\text{p53}}^{\left(m\right)}P_{B\geq x_{C}-m}\mathbf{1}_{m+1},\text{ }n=y_{C},y_{C}+1,\cdots,x_{C}-1,x_{C}; \\ nd_{\text{A}}\mathbf{1}_{n}+\sum_{m=0}^{n-1} g_{\text{p53}}^{\left(m\right)}P_{B\geq n-m}\mathbf{1}_{m+1},\text{ }n=x_{C}+1,x_{C}+2\cdots,C. \end{matrix}\right.$$

In brevity, the involved vectors can be expressed as

$$\mathbf{W}_{d_{\text{A}}}^{T}=\left[\left[0,\cdots,0\right],\cdots,\left[0,\cdots,0\right],\left[0,\cdots,0,\left(x_{C}+1\right)d_{\text{A}},0,\cdots,0\right],\right.\left.\cdots,\left[0,\cdots,0,Cd_{\text{A}}\right]\right]$$

The formulation for calculating the moments of the FPT is the same as [Eq. S17](#eqs17).

Numerical results for mean FPT and timing variability are shown in [Fig. S8](#figs8).

From [Figs S6](#figs6)*A*, [S7](#figs7)*A* and [S8*A*](#figs8), we can observe that the absorbing domains specified above are smaller than the one in [Fig. S3*A*](#figs3). [Figures S6](#figs6), [S7](#figs7), [S8](#figs8) demonstrate that fluctuations in event threshold can affect the mean FPT that the regulated protein reaches a certain fluctuating threshold for the first time, and event threshold can impact the timing variability. We also observe that for a high event threshold, fluctuations in event threshold can improve the event respond and shorten the time of FPT. The corresponding variability tendency in the timing, which raises the timing precision, also makes this result clear.

**Fig. S8 Characteristic of the curve for timing mean or timing variability vs event threshold in the case of absorbing domain** . (A) Schematic for PFT when an absorbing domain is defined by with $x_{\text{C}}>y_{C}$. (B) Timing mean as a function of event threshold for two different kinds of thresholds, where there is a crossing point $P$ which the same mean with the [Fig. S6*B*](#figs6). (C) Timing variability as a function of event threshold for two different kinds of thresholds, where two empty circle represent the crossing point of two curves, denoted by $RP_{1}$ and $RP_{2}$. In (B) and (C), the parameter values are set as $g_{\text{p53}}=5$, $d_{\text{p53}}=1$, $b=1$, $x_{\text{C}}=10$, $y_{\text{C}}=5$, and $p53_{\max}=50$. The threshold is set as $A_{\text{thresold}}=g_{\text{A}}/d_{\text{A}}$, where keeps always $d_{\text{A}}=1$, thus $A_{\text{thresold}}=1\sim15$ is decided by changing $g_{A}$. If the fixed threshold is set as $A_{\text{threshold}}=10$, the fluctuating threshold corresponds to $g_{\text{A}}=10$ and $d_{\text{A}}=1$.

# 4 Effect of timescales on the timing event

In this section, we investigate the effects of timescales on timing precision and mean FPT. For simplicity, we consider the simplest case i.e., the absorbing domain is (referring to [Fig. S3*A*](#figs3)). We firstly define the timescale in event timing. If the production and degradation rate of protein $p53$ or $A$ are simultaneously enlarged by $\alpha_{\text{p53}}$ or $\alpha_{\text{A}}$ times, the factor $\alpha_{\text{p53}}$ or $\alpha_{\text{A}}$ is defined as the timescale of protein $p53$ or $A$. The size of factor $\alpha_{\text{p53}}$ or $\alpha_{\text{A}}$ usually affects fluctuations in protein $p53$ or $A$. Now, we derive analytical results on the effects of timescale factors.

Based on the definition of timescale and by matrix $\mathbf{M}$in [Eq. S31](#eqs31), and by simultaneously enlarging the production and degradation rate of protein $p53$ or $A$, we have

Thus $\mathbf{M}$ can be rewritten as $\mathbf{M}=\alpha_{\text{p53}}\mathbf{M}_{1}+\alpha_{\text{A}}\mathbf{M}_{2}$, where $\mathbf{M}_{1}=diag\left(\mathbf{D}_{1}^{\text{p53}},\mathbf{D}_{2}^{\text{p53}},\cdots,\mathbf{D}_{C}^{\text{p53}}\right),$

$\mathbf{M}_{2}=\left[\begin{matrix} \mathbf{D}_{1}^{\text{A}} & \mathbf{U}_{1} & & & & \\ \mathbf{L}_{1} & \mathbf{D}_{2}^{\text{A}} & \mathbf{U}_{2} & & & \\ & \mathbf{L}_{2} & \mathbf{D}_{3}^{\text{A}} & \mathbf{U}_{3} & & \\ & & \ddots& \ddots& \ddots& \\ & & & \mathbf{L}_{C-2} & \mathbf{D}_{C-1}^{\text{A}} & \mathbf{U}_{C-1} \\ & & & & \mathbf{L}_{C-1} & \mathbf{D}_{C}^{\text{A}} \end{matrix}\right]$ (S58)

with , , $i=1,2,\cdots,C.$

The inverse matrix of $\mathbf{M}$ is then $\mathbf{M}^{-1}=\left(\alpha_{\text{p53}}\mathbf{M}_{1}+\alpha_{\text{A}}\mathbf{M}_{2}\right)^{-1}$. Regarding [Eq. S19](#eqs19), we can deduce the following expressions

Thus, the variability in the timing is calculated according to

(S59)

with $\gamma=\alpha_{\text{A}}/\alpha_{\text{p53}}$. [Eq. S59](#eqs59) shows how timing variability depends on the rate $\gamma$ between the timescales of proteins $p53$ and $A$. This dependence implies that variability timing does not depend on the timescale of proteins $p53$ or $A$, but depends on rate $\gamma$.

# 5 FPT statistics in the case that burst size follows Poisson distribution

The above minimal model considers that burst size $B$follows a geometric distribution. Here, we consider that $B$follows a Poisson distribution. We focus on how fluctuations affect mean FPT and timing variability. Number results are shown in [Figs. S9](#figs9) and [S10](#figs10). We observe that these results are analogous to those obtained above, implying that burst size distributions have little influence on mean FPT and timing variability.

For other three cases where burst size follows a Poisson distribution, the mean FPT and timing variability also have absorbing domains and change trends similar to those in the above respective three cases, as the fluctuating threshold increases. Numerical results are shown in [Figs. S11](#figs11), [S12](#figs12), and [S13](#figs13).

**Fig. S9** **Characteristic of the curve for timing mean or timing variability vs event threshold in the case of absorbing domain** , where burst size follows a Poisson distribution. (A) Timing mean as a function of event threshold for two different kinds of thresholds, where the inset shows FPT distributions for a specific event threshold (indicated by empty circles) corresponding to $A_{\text{thresold}}=10$. (B) Timing variability as a function of event threshold for two different kinds of thresholds, where the empty circle (denoted by $RP$) represents the crossing point of two curves and the responding threshold is $A_{\text{thresold}}\approx8.8$. And stars represent the critical threshold that makes timing variability reach the minimum. Variability timing for the fixed threshold is minimum when $A_{\text{thresold}}=6$, but variability timing for the fluctuating threshold is minimum, when $A_{\text{thresold}}=7$. In (A) and (B), the parameter values are set as $g_{\text{p53}}=5$, $d_{\text{p53}}=1$, $b=1$, and $p53_{\max}=40$. The threshold is set as $A_{\text{thresold}}=g_{\text{A}}/d_{\text{A}}$, where keeps always $d_{\text{A}}=1$, thus $A_{\text{thresold}}=1\sim15$ is decided by changing $g_{A}$. The other parameters of FPT distribution in the inset are $g_{\text{A}}=10$, $d_{\text{A}}=1$, and $A_{\text{thresold}}=10$.

**Fig.S10 Characteristic of the curve for timing mean or timing variability vs event threshold in the case of absorbing domain** . (A) Timing mean as a function of the burst size, where the inset shows a partial enlarged diagram. (B) Timing variability as a function of the burst size, where $RP$ represents a critical point for which the corresponding mean burst size is $b\approx1.2$. Stars represent the critical mean burst size that makes timing variability reach the minimum, which is respectively, $b\approx2.6$ for the fixed threshold, and $b\approx2.8$ for the fluctuating threshold. In (A) and (B), the parameter values are set as $g_{\text{p53}}=5$, $d_{\text{p53}}=1$, $g_{\text{A}}=10$, $d_{\text{A}}=1$, and $p53_{\max}=40$. The range of mean burst size is set as $b=0.1\sim8$, and the threshold is set as $A_{\text{thresold}}=10$.

**Fig. S11 Characteristic of the curve for timing mean or timing variability vs event threshold in the case of absorbing domain** , where burst size follows a Poisson distribution. (A) Timing mean as a function of event threshold, where the inset shows a partial enlarged diagram. (B) Timing variability as a function of event threshold, where the two empty circles represent the crossing point of two curves, denoted by $RP_{1},RP_{2}$. And stars represent the critical threshold that makes the timing variability reach the minimum, that is $A_{\text{thresold}}=6$, for the fixed threshold, but $A_{\text{thresold}}=1$, for the fluctuating threshold. Burst size follows a Poisson distribution in (A) and (B). In (A) and (B), the parameter values are set as $g_{\text{p53}}=5$, $d_{\text{p53}}=1$, $b=1$, $x_{C}=5$, and $p53_{\max}=50$. If the fixed threshold is set as $A_{\text{threshold}}=10$, the fluctuating threshold corresponds to $g_{\text{A}}=10$ and $d_{\text{A}}=1$, which the threshold is set as $A_{\text{thresold}}=g_{\text{A}}/d_{\text{A}}$, where keeps always $d_{\text{A}}=1$.

**Fig. S12 Characteristic of the curve for timing mean or timing variability vs event threshold in the case of absorbing domain** , where burst size follows Poisson distribution**.** (A) Timing mean as a function of event threshold, where the inset shows a partial enlarged diagram. (B) Timing variability as a function of event threshold, where the two empty circles represent the crossing point of two curves, denoted by $RP_{1},RP_{2}$.And stars represent the critical threshold that makes timing variability reach the minimum, that is, $A_{\text{thresold}}=6$, for the fixed and fluctuating threshold. Burst size follows a Poisson distribution in (A) and (B). In (A) and (B), the parameter values are set as $g_{\text{p53}}=5$, $d_{\text{p53}}=1$, $b=1$, $y_{C}=5$, and $p53_{\max}=50$. If the fixed threshold is set as $A_{\text{threshold}}=10$, the fluctuating threshold corresponds to $g_{\text{A}}=10$ and $d_{\text{A}}=1$, which the threshold is set as $A_{\text{thresold}}=g_{\text{A}}/d_{\text{A}}$, where keeps always $d_{\text{A}}=1$.

**Fig. S13 Characteristic of the curve for timing mean or timing variability vs event threshold in the case of absorbing domain** , where burst size follows Poisson distribution. (A) Timing mean as a function of event threshold, where the inset shows a partially enlarged diagram. (B) Timing variability as a function of event threshold, where the two empty circles represent the crossing point of two curves, denoted by $RP_{1},RP_{2}$. And stars represent the critical threshold that makes timing variability reach the minimum, that is, $A_{\text{thresold}}=6$ for the fixed threshold, but $A_{\text{thresold}}=8$ for the fluctuating threshold. Burst size follows a Poisson distribution in (A) and (B). In (A) and (B), parameter values are set as $g_{\text{p53}}=5$, $d_{\text{p53}}=1$, $b=1$, $x_{C}=10$, $y_{C}=5$, and $p53_{\max}=50$. If the fixed threshold is set as $A_{\text{threshold}}=10$, the fluctuating threshold corresponds to $g_{\text{A}}=10$ and $d_{\text{A}}=1$, which the threshold is set as $A_{\text{thresold}}=g_{\text{A}}/d_{\text{A}}$, where keeps always $d_{\text{A}}=1$.

# FPT problem involved cell division

**Fig. S14 Schematic diagram of FPT problem involved cell division in the fluctuating threshold case**, where $t_{\text{D}}$ represents a cell cycle. (A) showing the apoptotic of protein p53 occurs before cell division. (B) showing the apoptotic of protein p53 and its daughter may happen after cell division.

# References

1. [Redner S. 2001. A Guide to First-passage processes. Cambridge: Cambridge University Press.](https://doi.org/10.1017/CBO9780511606014)

1. [Iyer-Biswas S, Zilman A. 2016. First-passage processes in Cellular Biology. Advances in Chemical Physics.](https://doi.org/10.1002/9781119165156.ch5) **[160](https://doi.org/10.1002/9781119165156.ch5)**[(Chap.5): 261-306.](https://doi.org/10.1002/9781119165156.ch5)

1. [Ghusinga KR, Dennehy JJ, Singh A. 2017. First-passage time approach to controlling noise in the timing of intracellular events.](https://doi.org/10.1073/pnas.1609012114) *[Proc. Natl. Acad. Sci. USA](https://doi.org/10.1073/pnas.1609012114)* **[114](https://doi.org/10.1073/pnas.1609012114)**[: 693-698.](https://doi.org/10.1073/pnas.1609012114)

1. [Van Kampen N. 2007.](https://www.sciencedirect.com/book/9780444529657/stochastic-processes-in-physics-and-chemistry) *[Stochastic process in physics and chemistry. 3rd ed](https://www.sciencedirect.com/book/9780444529657/stochastic-processes-in-physics-and-chemistry)*[. North-Holland, Amsterdam.](https://www.sciencedirect.com/book/9780444529657/stochastic-processes-in-physics-and-chemistry)
